# The history of the formation of the Pan African paediatric surgical Association (PAPSA)

**DOI:** 10.1007/s00383-018-4248-y

**Published:** 2018-03-27

**Authors:** Heinz Rode, Julius Kyambi, Kokila Lakhoo

**Affiliations:** 1 Red Cross Children’s Hospital, University of Cape Town, Cape Town, South Africa; 2 Jomo Kenyatta Hospital, University of Nairobi, Nairobi, Kenya; 3 Department of Paediatric Surgery, University of Oxford and Oxford University Hospitals NHS Trust, Oxford, OX39DA UK

**Keywords:** Pan African, Association of Paediatric Surgeons, History of paediatric surgery

## Abstract

Political change in the 1990s in Southern Africa influenced the long awaited formation of a children’s surgical association in Africa. Here, within is the historical account of the formation of the Pan African Association of Paediatric Surgeons (PAPSA). The basis of the argument for the formation of PAPSA was that paediatric surgeons from Africa have a wealth of experience and special knowledge of diseases affecting children in the African continent which differ substantially from those encountered in other regions of the world. This historical account is mainly from documentation by 2 of the organisations founding members.

**Electronic supplementary material:**

The online version of this article (10.1007/s00383-018-4248-y) contains supplementary material, which is available to authorized users.

Political developments in Southern Africa during the early 1990s excited the World. Talks were being held for the release of Mr. Mandela and the opportunity arose for change. As this ‘wind of change’ swept across Africa, two leading paediatric surgeons from South Africa, namely, Prof. S. Cywes and Prof. M. R. Q. Davies, proposed that colleagues in Africa should be approached with the view of establishing a representative African Paediatric Surgical Association or group (Supplementary Appendixes 1 and 2).

During the 38th British Association of Paediatric Surgeons (BAPS) conference in Budapest 1991, Prof. H. Rode and Prof. S. Hamdy took the concept further and discussed this “embryo concept” of an Association representing Africa with nine paediatric surgeons from Africa who attended the 38th BAPS meeting. The core group were: P. Beale, R. Brown, S. Cywes, G. P. Hadley and H. Rode from South Africa; H. Hamdy from Egypt; A. Hesse from Ghana; M. Oliver from Zimbabwe and P. Carneiro from Tanzania (Fig. 1).

**Fig. 1:**
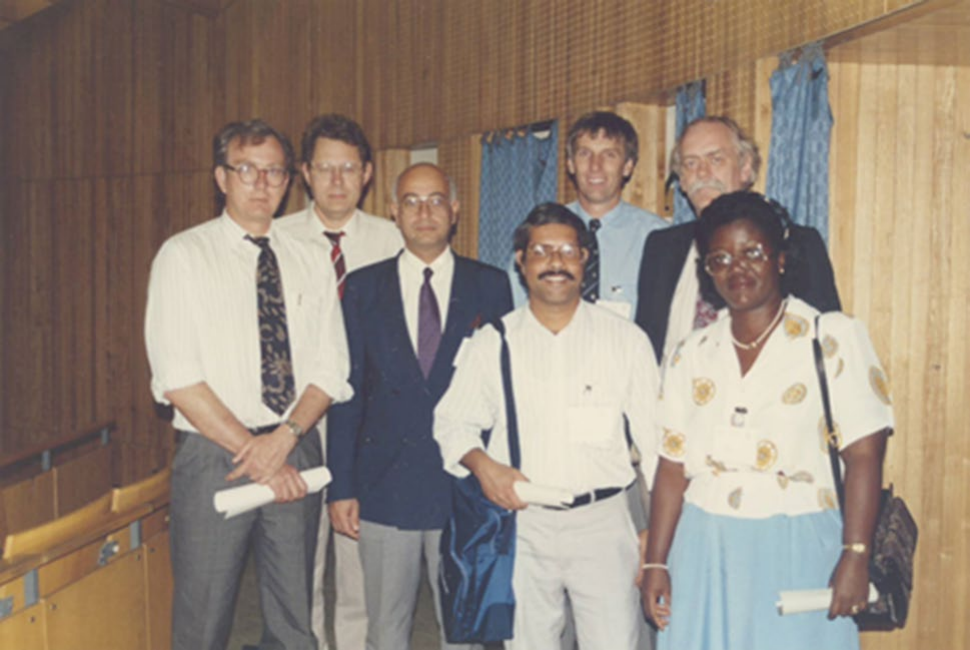
L-R: P. Beale, H. Rode, H. Hamdy, P. Carneiro, R. Brown, G. P. Hadley, A. Hesse. The founding paediatric surgeons who met in Budapest during the 38th BAPS conference

The group assembled for the first time after the last session of the second day to discuss the value of international meetings as well as the creation of one which could represent the interests of Africa, a mere 4 months from the conception of such a possibility. The basis of the argument for this was that paediatric surgeons from Africa have a wealth of experience and special knowledge of diseases affecting children in the African continent which differ substantially from those encountered in other regions of the world. The formation of such an association could have a fourfold effect. First, the organisation would establish principles for the surgical care for children in Africa. Second, it act as a forum for training and encouragement in the study of paediatric surgery. Third, it form a basis of regular meetings for the interchange of ideas and sharing of knowledge and expertise and, finally, to encourage research on topics that are relevant to the African continent. The ultimate goal has been to improve surgical services to the children of Africa. This concept was further developed amongst the group during a subsequent paediatric surgical meeting in Prague in 1991.

As the fledging idea needed international recognition and support, Prof. Rode wrote to the President of the World Federation of Associations of Paediatric Surgeons (WOFAPS), Prof. Keith Aschcraft, who responded on the 30 July 1991 stating “It was the consensus of the Executive Committee that such an alliance would be a very positive thing in the health and well-being of African children. We would therefore be pleased to help in any way that we could.” These sentiments were prophetic in that WOFAPS gave their full and unending support during the development phases of the as yet to be established Association (Supplementary Appendix 3).

Profs. H. Rode, and S. Hamdy constructed a draft proposal in the form of “Call for Input” which was sent to surgeons in Africa on the 17 October 1991 for ideas and to test the waters for such a proposal (Supplementary Appendix 4). This concept was fully endorsed by African paediatric surgeons who were mainly from Kenya, Tanzania, Zimbabwe, Ghana, Malawi, Egypt, South Africa and the Northern Mediterranean coast of Africa. Support from Prof. J. Kyambi, from the Kenyatta National Hospital in Nairobi was crucial and played a significant role in the milestone of the organisation as he was well known in the field of children’s surgery in Africa. Also the political climate change in South Africa allowed for engagement with South African surgeons who were previously wrapped up in political sanctions (Supplementary Appendix 5).

In December 1991, the Egyptian Association also gave their support for the fledging Association which now had support from both North and South of the equator. On 28 June 1992, Prof. Hamdy received communication and an invitation from the International College of Surgeons (Prof. Refaat Kamel) for the still-to-be Association to become an affiliate member. This further supported the widespread enthusiasm for the proposed new African Paediatric Surgical Association.

Additional support for the proposal was received from paediatric surgeons from North Africa especially from Egypt, Libya, and Sudan. The suggestion was also tabled that the first meeting of the Association should coincide with the International College of Surgeons meeting scheduled for 28 November 1992 in Cairo. Unfortunately, this did not materialise as the basic ground work for the new Association was still incomplete. It took another 2 years before the establishment of the Pan African Paediatric Surgical Association (PAPSA) could be celebrated.

Further support was given by Prof. J. Boix-Ochoa, secretary/treasurer of WOFAPS, on 16 October 1992 and Prof. Ashcraft also offered to assist in organizing a scientific conference in Nairobi during the latter half of 1993 or early in 1994. Throughout the initial phases, the vital role played by WOFAPS must be emphasised in their unyielding academic and financial support for the concept.

It was importance that the Association be owned by surgeons from Africa and not be seen as solely South African-based. It became clear that an Association representing Africa would be a feasible endeavour and was long overdue. However, from early on, reservations were expressed of linking PAPSA with other Associations and the prevailing idea was for the Association to be non-aligned and independent. This did not, however, exclude combined meetings with the reservation that PAPSA would only participate as an independent Association.

As the current methods of communication at that time (telephone, fax and postal services) were slow and cumbersome, opportunities were sought to meet for further discussions, where paediatric surgeons assembled during regional conferences. As this initiative was new for paediatric surgery in Africa, all efforts were directed towards inclusiveness. Two further opportunities presented when paediatric surgeons from Africa assembled at regional conferences. Many attended the South African Paediatric Surgical Association’s biannual meeting in the Transkei, South Africa, in July 1992, and fruitful discussions ensued, especially with surgeons from Kenya and Ghana. It was during a meeting of the International Federation of Surgical Colleges in Cape Town that Prof. Rode spoke with Prof. O. Ayaji, the President of the College of Surgeons of West Africa. Long and fruitful discussions followed regarding the concept and implementation of the proposed Association which was seen as a strengthening of the role paediatric surgery could play in improving the health of its children. Another step forward with support from West Africa.

A suitable geographical place on the Continent of Africa had to be found, where a meeting of paediatric surgeons could take place and the idea further developed. Nairobi being in a central position in Africa, was considered as a possible venue for the launching of the Association. Such an opportunity presented itself during the ano-rectal workshop held by Prof. A. Pena in November 1992, at the Jomo Kenyatta Hospital, Nairobi, under the auspices of Prof. J. Kyambi from the University of Nairobi.

The momentum was unstoppable. The ano-rectal workshop in Nairobi in November 1992 brought 30 paediatric surgeons from East, Central and Southern Africa together. Prof. J. Kyambi played a pivotal role in all the negotiations for the formation of PAPSA. Delegates met to discuss and finalise the concept and establishment of an African Association for Paediatric Surgery. Again, the desire was expressed to form an Association amongst people with a common goal and regional interest and that membership should encompass all paediatric surgeons from Africa. A steering committee was constituted to oversee the initiation of the Association on the 3 November 1992. South Africa was reluctant to drive the Association and was, therefore, pleased when very competent people from Kenya, Tanzania, and Ghana were elected to the steering committee to oversee the formation of the Association. A strong request was made that all future official committees should be representative of the continent as a whole. Prof. Boix-Ochoa was informed on the 30 November about the developments, the elected steering committee and the immediate goals of the Association to be formed in the near future. The centre of Africa thus became the birth place of PAPSA.

Finally, after many months of anxiety, ground work preparation, consultations, letters, faxes and telephone calls, the letter sent out from the interim secretary on 15 July 1993 read “It is with great pleasure that we announce the formation of the Pan African Paediatric Surgical Association (PAPSA) and advise you of its Inaugural Meeting scheduled to be held in Nairobi from 9 to 11 March 1994.”

The minutes of the first meeting to consider the establishment of a Pan African Surgical Association in November 1992 is appended (Supplementary Appendix 6). The office bearers were Interim Chairman: Prof. J. Kyambi, Kenya; Vice chairman: Prof. J. Shija, Tanzania; Secretary: Prof. H. Rode, South Africa and two additional members: Dr. A. Hesse, Ghana; Dr. Ndingo, Kenya. Resolution: that PAPSA should be established within 1 year and that the concept of PAPSA be promoted at all forums. The World Federation of Associations of Paediatric Surgeons endorsed the formation of PAPSA. Four immediate goals were identified:

1. to promote and maintain the highest clinical and ethical standards;
2. to encourage and arrange regular meetings for the interchange of ideas and sharing of knowledge and expertise;
3. foster professional relations with Paediatric surgeons throughout the world;
4. to promote the practice, research and the advancement of study in Paediatric Surgery.

Outstanding were the final structure and function of the Association and the formalization of the constitution. On 25 May 1993, Profs. Kyambi and Prof. Rode posted a letter of invitation to surgeons in Africa to become members of the fledging Association (Supplementary Appendix 7). The response was overwhelming. An important next step was to constitute a proposed constitution as was legally required. Many African colleagues contributed to the final constitution of the Association, especially Profs. Shija, Davies, Cywes, Kyambi, Rode, Oliver, and Hamdy.

In correspondence with the South African Department of National Health and Population Development on 13 November 1992, the interim secretary again defined the mission of PAPSA: “To serve the children of Africa, to encourage the study of paediatric surgery, to promote and maintain the highest clinical standards, to encourage research within Africa and to have regular conferences for the interchange of ideas and sharing of knowledge and expertise”.

On 17 February 1993, a formula was presented regarding the first meeting scheduled for 9–11 March 1994 in Nairobi, with the theme “Towards the improvement of paediatric surgical care for the children of Africa”.

This was soon followed by a meeting of interested people during the BAPS conference in Manchester in July 1993 when the Association was discussed with the President of WOFAPS, Prof. Dr. Wolfgang Maier, who expressed his delight about the founding of PAPSA. In attendance at the meeting were Profs. J. Kyambi, J. Shija, S. Hamdy, H. Rode, and Dr. L. Ekiabi by invitation.

It was resolved to finalise the constitution for ratification and to solicit membership for the fledging Association from surgeons in Africa. The inaugural meeting was scheduled for 9–11 March 1994 and five invited guests were identified as a measure of the widespread acceptance of the concept of an independent Association representing the interests of Africa. These were Profs. W. Maier (WOPCES), J. Boix-Ochoa (WOFAPS), J. Kyambi (PAPSA interim President), K. Ashcraft (Past President APSA), and J. J. Corkery (President BAPS). They were individually acknowledged because of their support and direct involvement in establishing PAPSA.

Despite all the logistical difficulties in the African continent, the concept was approved; an interim committee formed; a constitution written; and the inaugural meeting of PAPSA scheduled for the 9–11 March 1994 in Nairobi with Prof. Kyambi as first Chairman and primary organiser. He deserved much credit for all his endeavours to form a fertile field for the initiative during the changing phase in Africa.

Information about PAPSA was posted on the 25 May 1993 to most of the established Paediatric Surgical Associations in the World and paediatric surgeons on the African continent. They all responded enthusiastically and gave their full support to the new Association. Summarizing their sentiments, Prof. Andrew Pinter wrote the following on 3 August 1993: " We are deeply convinced that the PAPSA will contribute not only to the maintenance of the highest clinical and ethical standards, but will promote the better understanding amongst the nations and people of Africa, and furthermore, it will strengthen the friendship amongst the paediatric surgeons of the world. Additional interest in the Association came from Francophone countries in West Africa, America, Europe, Canada, England, Scotland, China and India, as well as many other centres in Africa. The widespread support was most encouraging and heart-warming.

Recognition of the important and crucial role surgery played on the African continent was not freely forthcoming from International bodies. The reservations WHO and UNICEF had about the position of paediatric surgery in Africa was mentioned in two letters dated 6 September 1993 and 3 March 1994. In 1993, Prof. Kyambi wrote “I am much too aware that the international bodies, that is UNICEF and WHO are not keen to support surgical programs. Somehow the concept they have is that surgery is too sophisticated for developing countries and they put their entire support for Paediatric programs, Mother and Child and Child Survival Programs. They forget that surgery is also geared to child survival. We still have a lot to do”. It took over 20 years for the international bodies such as WHO to understand the need of the surgical child through the launch of the Lancet Commission in 2015 [1].

In 1994, Prof. K. Mukelabai, Regional Adviser for Primary Health Care, wrote to Prof. Kyambi “It is important to remind all the eminent paediatric surgeons at your meeting that the major causes of childhood mortality and morbidity in Africa are all preventable …. Both medical and surgical complications of these diseases are difficult to treat in the late stages”. On a very positive note, however, acknowledgment of the important role surgery played during childhood was emphasised by Prof. F. Bassani, Deputy director of UNICEF Geneva, in a letter to Prof. S. Cywes on the 9 September 1993 in which he categorically stated that UNICEF is committed to help the African Child towards a better future and pledges their participation towards the foundation of the new Pan African Paediatric Association. The regional UNICEF office in Nairobi assisted PAPSA with a sum of USD 3000.

As this was a new initiative for Africa, sponsorship was initially difficult to obtain. The interim secretary had tried his best, pleaded, threatened, prayed, and cursed, given up hope, but eventually, with the help and encouragement of all involved with this venture, succeeded in securing financial backing. The first budget submitted was: Expenditure USD 2577.10 and Income USD 19,342.00. Very generous and essential financial support of USD 10,000 was received from WOPSEC (Prof. C. Ghinelli); Department of Foreign Affairs South Africa SAR 11,844; South African Association of Paediatric Surgeons SAR 6000; UNICEF USD 3000; ten pharmaceutical firms (USD 1730.00); SAIDIA SAR 6076; and a personal contribution from Prof. H. Rode, SAR 5642.

The Centre for Postgraduate Studies of the University of Cape Town was appointed as congress organisers. The centre’s professional attitude and guidance towards the financial concerns, organisational structure, and personnel communications directly ensured the eventual success of the conference. The British Association of Paediatric Surgeons offered two Heinz Scholarships to PAPSA following the Inaugural meeting and two trainee surgeons, Drs. Q. Ossenou from Coter de’Ivoire and J. N. Muturi from Kenya, were the successful candidates.

The final announcement was posted in January 1994. The organizing committee of the meeting was Prof. J. Kyambi, J. M. Ngungu, S. A. Safwat, N. N. Wachira, G. C. Anangwe, S. M. Barrack, P. Ochola-Abila, and Lt Col(Dr)BN Waitara. All of them subsequently played leading roles in PAPSA for many years to come.

The Inaugural meeting of PAPSA was held on 9–11 March 1994 at the historical Hotel Inter-Continental in the centre of Nairobi. The theme of the congress was “Towards the improvement of paediatric surgical care for the children of Africa” [2].

The official opening of the conference was an illustrious occasion. The Master of Ceremony was Mr. P. Ochola-Abila and the welcoming address was given by Prof. Kyambi (Interim President) followed by addresses by executives from Ministry of Health, Republic of Kenya (Hon. J. Angatia), SAIDIA (Mr. B. Kliplagat), WOFAPS (Prof. C. Ghinelli), WOFAPS (Prof. W. Maier), Minister of Health and Director of Medical Services (Prof. G. B. A. Okelo). Three keynote addresses were given by Prof. S. Cywes from South Africa, Prof. J. Shija from Tanzania and Prof. Nabham Kadah from Egypt. Prof. J. J. Corkery, President of BAPS, was the after conference dinner speaker. Other important delegates were Dr. E. Kruger (South African Embassy), Prof. Makelabai (UNICEF), Prof. Chube (WHO), and Dr. R. Leakey (SAIDIA).

The minutes of the first Exco and inaugural meetings of PAPSA on the 9 and 11 March 1994, respectively, are appended (Supplementary Appendix 8 and 9). Prof. J. Kyambi was the first President with Prof. J. Kaddah Vice president and Prof. H. Rode as Honorary Secretary/Treasurer. Ten members were elected: S. Bankole (Ivory Coast), S. Cywes (South Africa), M. Davies (South Africa), H. Hamdy (Egypt), A. Hesse (Ghana), S. Mutumba (Uganda), P. Nmandu (Nigeria), M. Oliver (Zimbabwe), A. Safwat (Kenya), and J. Shija (Tanzania). More than 80 surgeons attended the 3-day conference (Fig. 2).

**Fig. 2:**
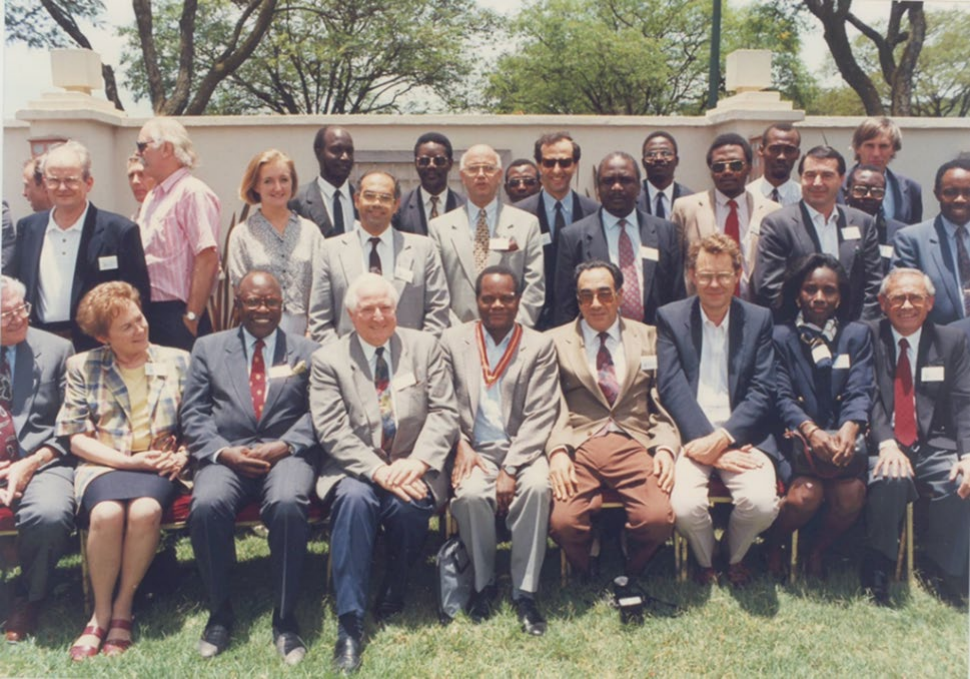
Delegates attending the inaugural meeting of the Pan African Paediatric Surgical Association 9–11 March 1994 Nairobi Kenya

The Francophone countries were not present at this stage of our evolution, although they have voiced an interest. It was also resolved that PAPSA should have biennial meetings with the venues rotating on a regional basis. The Association’s constitution was ratified after minor changes from the original submission. Of great importance was that there would always be geographic representation.

Dr. A. S. Safwat was the treasurer for the first meeting and his hard work and enthusiasm has made organizing the meeting relatively stress free. The conference was successfully concluded with a budget of SAR 82,437.86. In addition, the financial invoice of the 9 February 1994 indicated the complexity of arranging the first meeting of the Association.

Many surgeons contributed to the planning of the first scientific program, as it had to be specific to the needs of Africa. These included Profs. M. Davies, J. Shija, H. Hamdy, S. Cywes, J. Kyambi, and executive members from other International Associations. The presentations all addressed surgical conditions commonly seen in Africa, including oncology, trauma, congenital abnormalities, infections, orthopaedic conditions, and paediatric surgical experiences in emerging countries. Debate was vigorous and informative and the keynote presentations were relevant to our problems. Delegates retired after three historical days of scientific deliberations, new friendships were made, while old ones were renewed and strengthened. Upon departure, there was a realisation that Africa now has an Association to benefit the children of Africa. PAPSA was born and it then needed hands to nurture, feed, and mature the child. The second meeting was scheduled for 1996 in Cairo, followed by the third meeting in Cape Town in 1998 (Table 1).

**Table 1:** PAPSA meetings held to date

Year	In association with	Venue
1994	World Federation of Associations of Paediatric Surgeons	Nairobi, Kenya
1996	Egyptian Paediatric Surgery Association (EPSA)	Cairo, Egypt
1998	South African Association of Paediatric Surgeons (SAAPS)	Cape Town, South Africa
2000	Cancelled	Abidjan, Côte d’Ivoire
2002	European African Paediatric Surgical Association	Cairo, Egypt
2004	International Society of Paediatric Oncology (SIOP)	Blantyre, Malawi
2005	Egyptian Paediatric Surgery Association (EPSA)	Alexandria, Egypt
2006	Kenyan Association of Paediatric Surgeons	Mombasa, Kenya
2008	Ghana Association of Paediatric Surgeons	Accra, Ghana
2010	Tanzanian Surgical Association (TSA)	Dar Es Salaam, Tanzania
2012	South African Association of Paediatric Surgeons (SAAPS)	Cape Town, South Africa
2014	Egyptian Paediatric Surgery Association (EPSA)	Cairo, Egypt
2016	Association of Paediatric Surgeons of Nigeria (APSN)	Lagos, Nigeria

In a letter to Mr. J. J. Corkery, President of BAPS, dated 2 June 1994, the secretary Prof. H. Rode reiterated the feeling amongst members that much help and enthusiasm would be needed to steer this Association on the charted course devised in Nairobi. Similar correspondence was sent to all who participated and supported in person, and to those who represented International Associations. Application was made in December 1994 for WOFAPS to become a member Association, which was subsequently granted.

The final reconciled financial statement regarding income and expenses was sent to Prof. C. Ghinelli (WOPSEC) on 14 July 1995 and the key role that WOPSEC played in the establishment of the Association was acknowledged.

Despite many obstacles, uncertainties and doubt the mission was accomplished. An independent Association was established, representing men and women who have dedicated their lives to the pursuit of improving the surgical care of African children, which they so richly deserved. PAPSA [2] has clearly defined goals with the most vital component of improving surgical care for the children of the African Continent. PAPSA has now two journals linked to it, namely, the Annals of Paediatric Surgery and the African Journal of Paediatric Surgery.

As so little was known about paediatric surgery on the continent, delegates considered it important to establish a basic data base for the Association, looking at manpower, facilities, patient numbers and profiles, surgical procedures, training facilities and mortality data. Unfortunately the means of communication were very basic, with telephone, letters and the occasional fax, which made it virtually impossible to obtain and distribute the information at the time. This would be for the future.

This is as accurate as we could reconstruct the periods preceding the establishment of PAPSA and the inaugural conference.

## Electronic supplementary material

Below is the link to the electronic supplementary material.

Supplementary material 1 (DOCX 1031 KB)

Supplementary material 2 (DOCX 476 KB)

Supplementary material 3 (DOCX 927 KB)

Supplementary material 4 (DOCX 1080 KB)

Supplementary material 5 (DOCX 1419 KB)

Supplementary material 6 (DOCX 913 KB)

Supplementary material 7 (DOCX 672 KB)

Supplementary material 8 (DOCX 734 KB)

Supplementary material 9 (DOCX 1122 KB)
