# Supplementary figures and images for: The history of the formation of the Pan African paediatric surgical Association (PAPSA)
Source: Pediatr Surg Int. 2018 Mar 27;34(5):499–504. doi: 10.1007/s00383-018-4248-y (PMC5899112; doi:10.1007/s00383-018-4248-y)

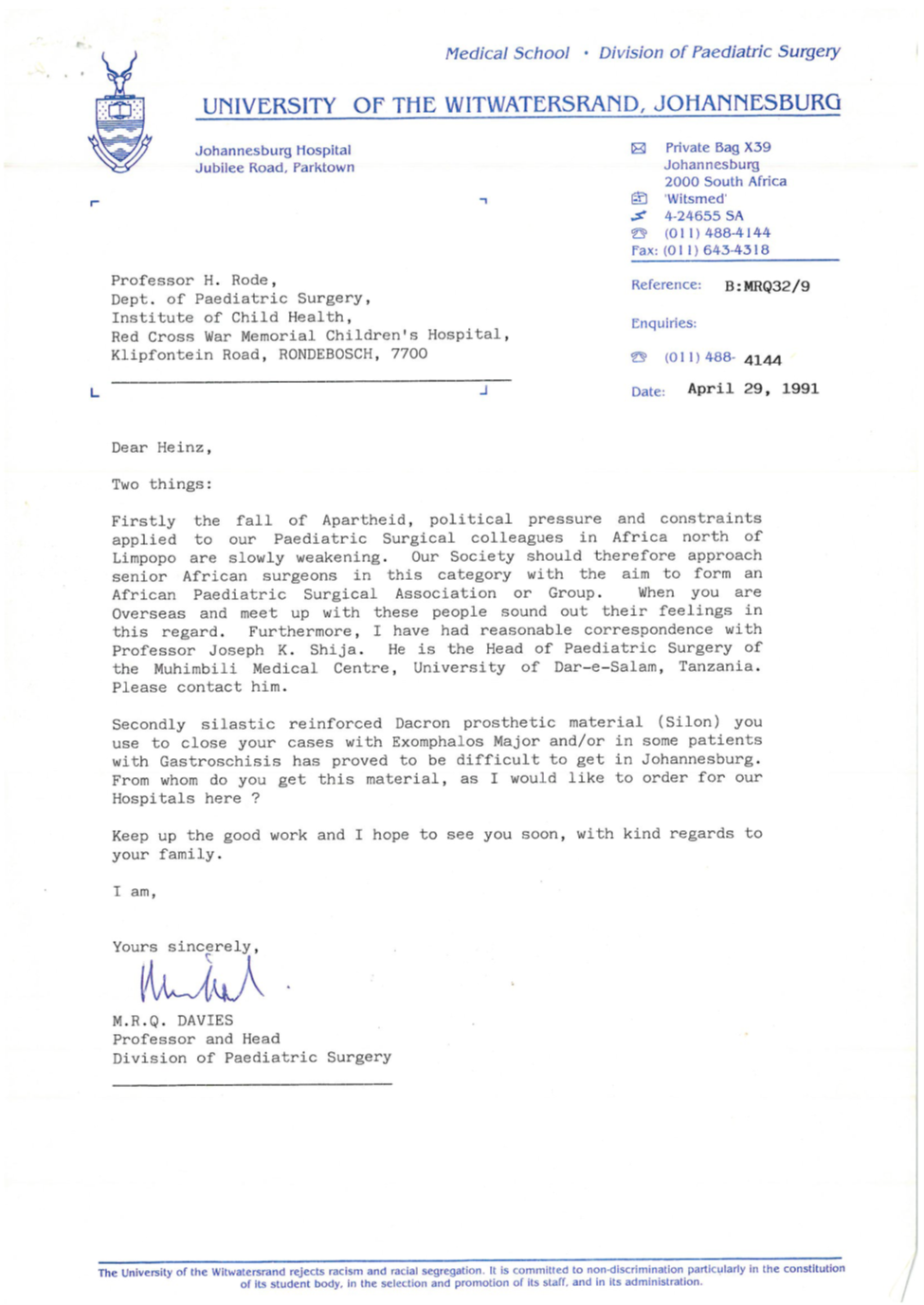

Supplement: Supplementary file 1 — Supplementary material 1 (DOCX 1031 KB) [file 383_2018_4248_MOESM1_ESM.docx]

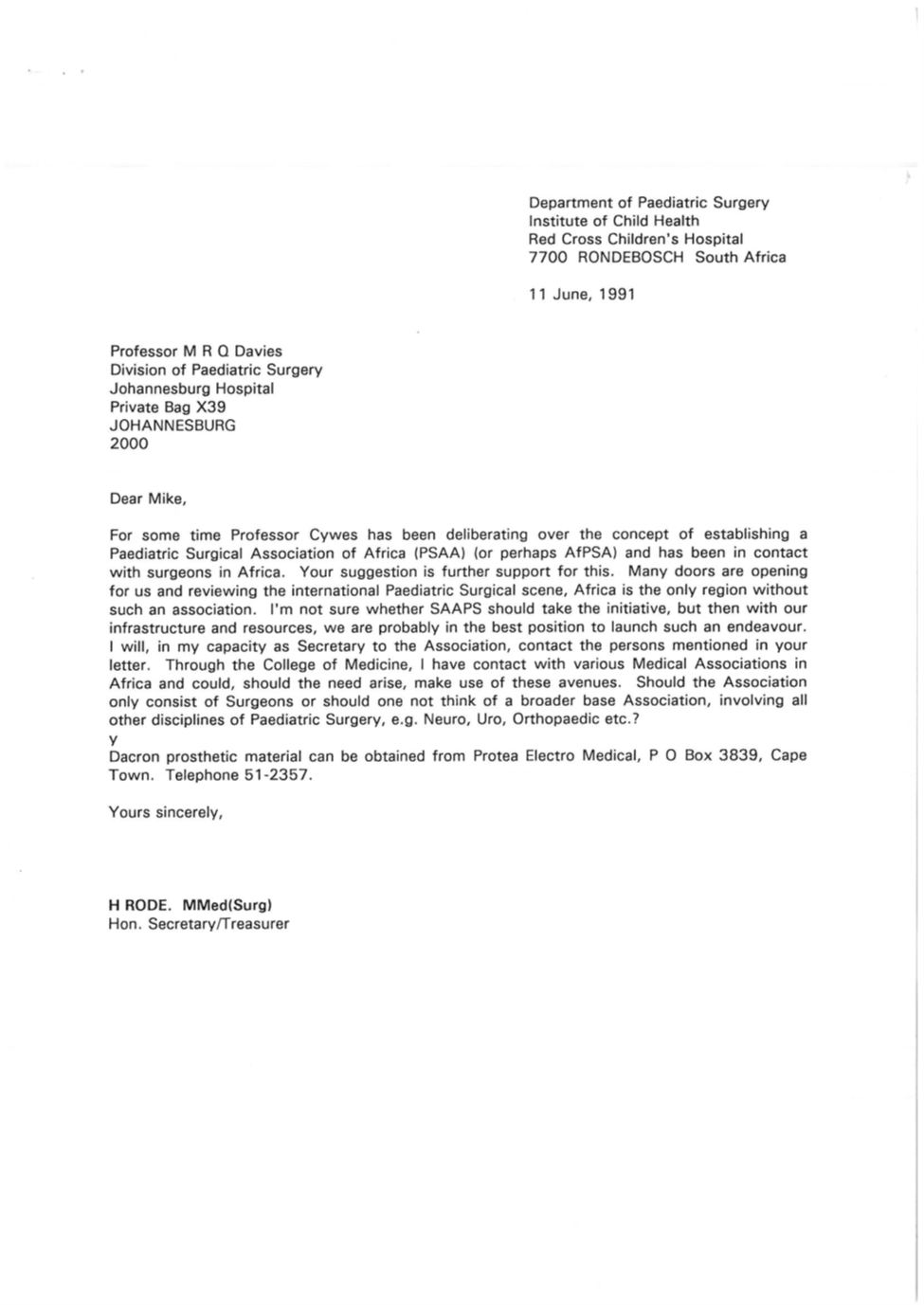

Supplement: Supplementary file 2 — Supplementary material 2 (DOCX 476 KB) [file 383_2018_4248_MOESM2_ESM.docx]

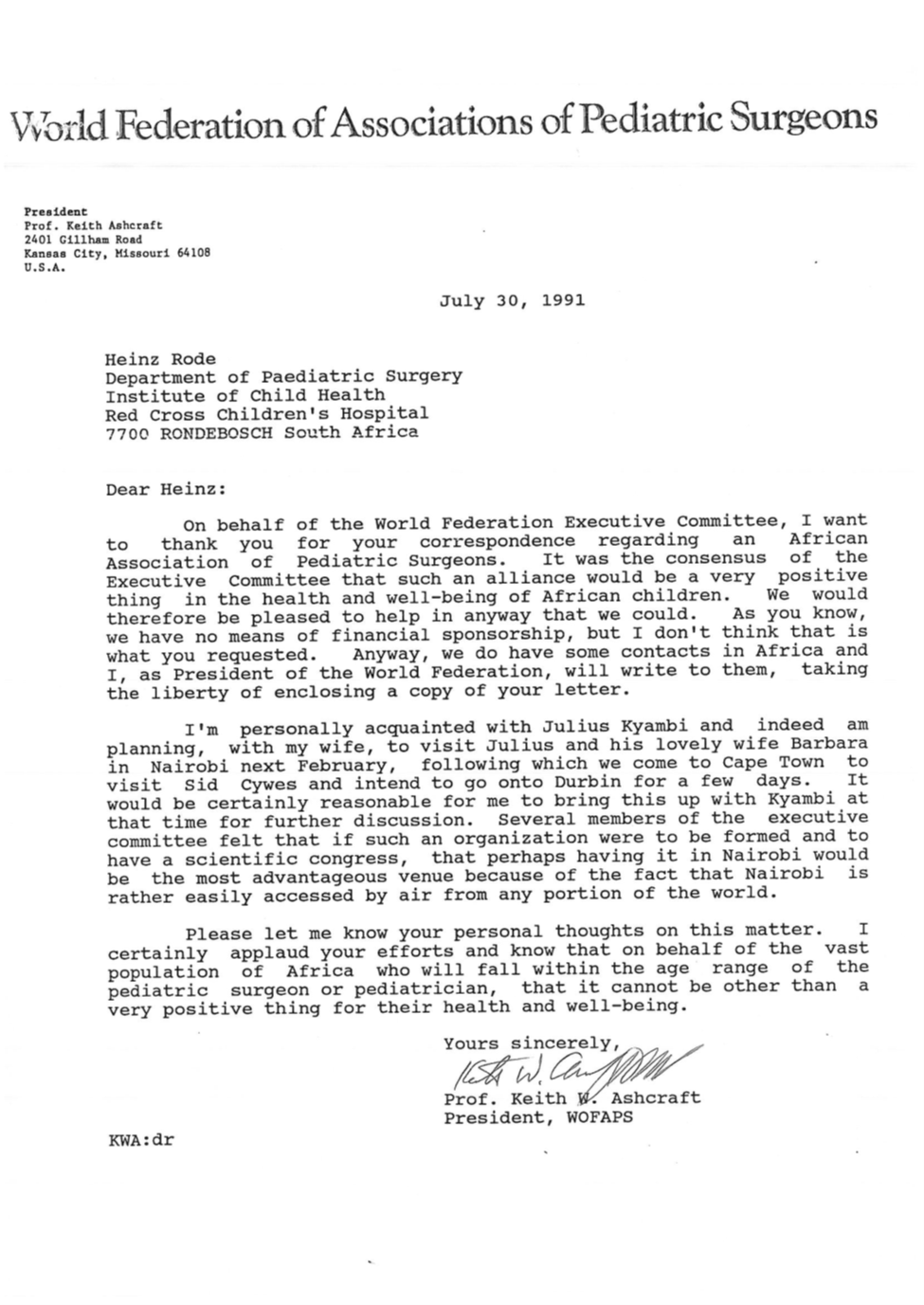

Supplement: Supplementary file 3 — Supplementary material 3 (DOCX 927 KB) [file 383_2018_4248_MOESM3_ESM.docx]

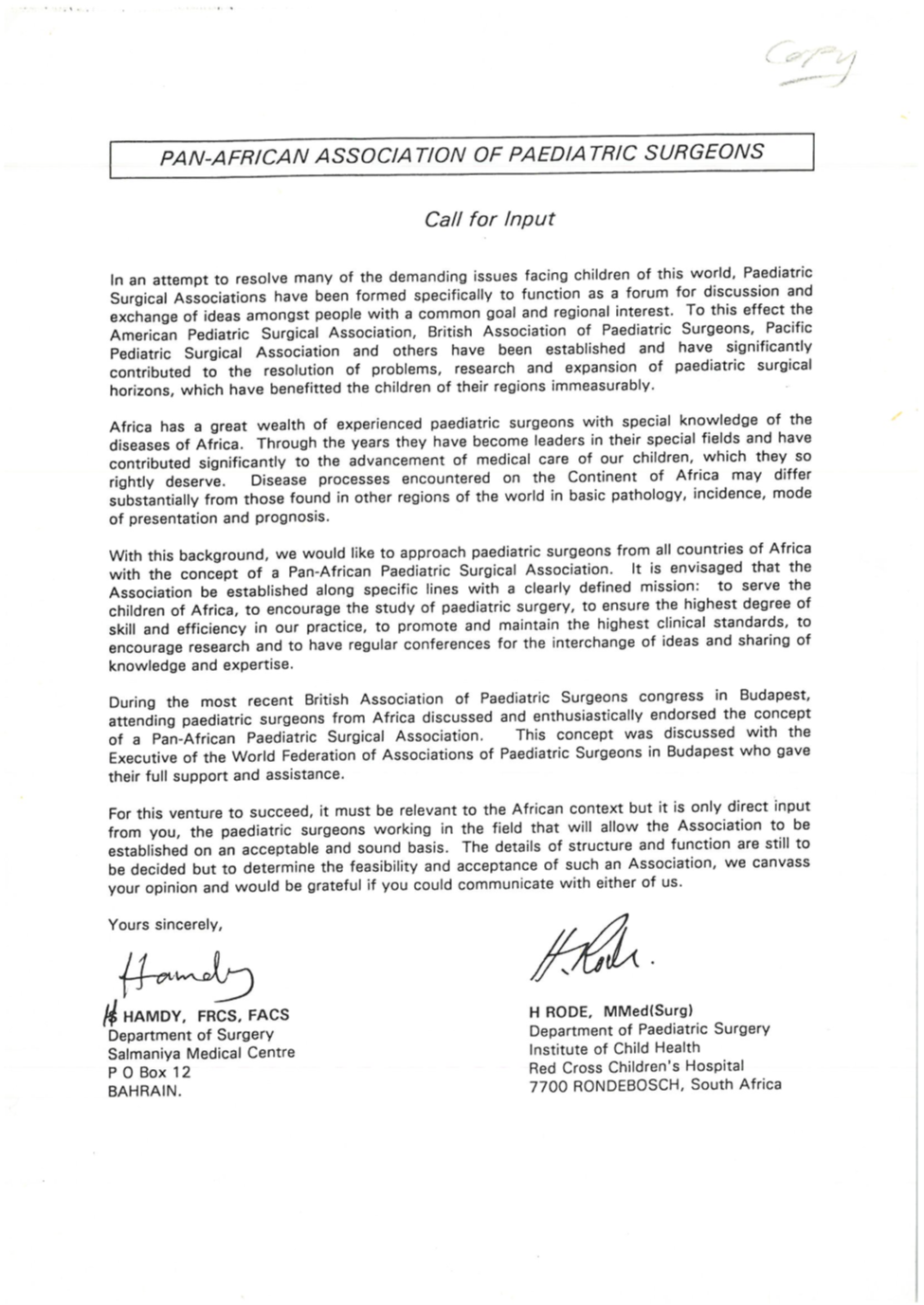

Supplement: Supplementary file 4 — Supplementary material 4 (DOCX 1080 KB) [file 383_2018_4248_MOESM4_ESM.docx]

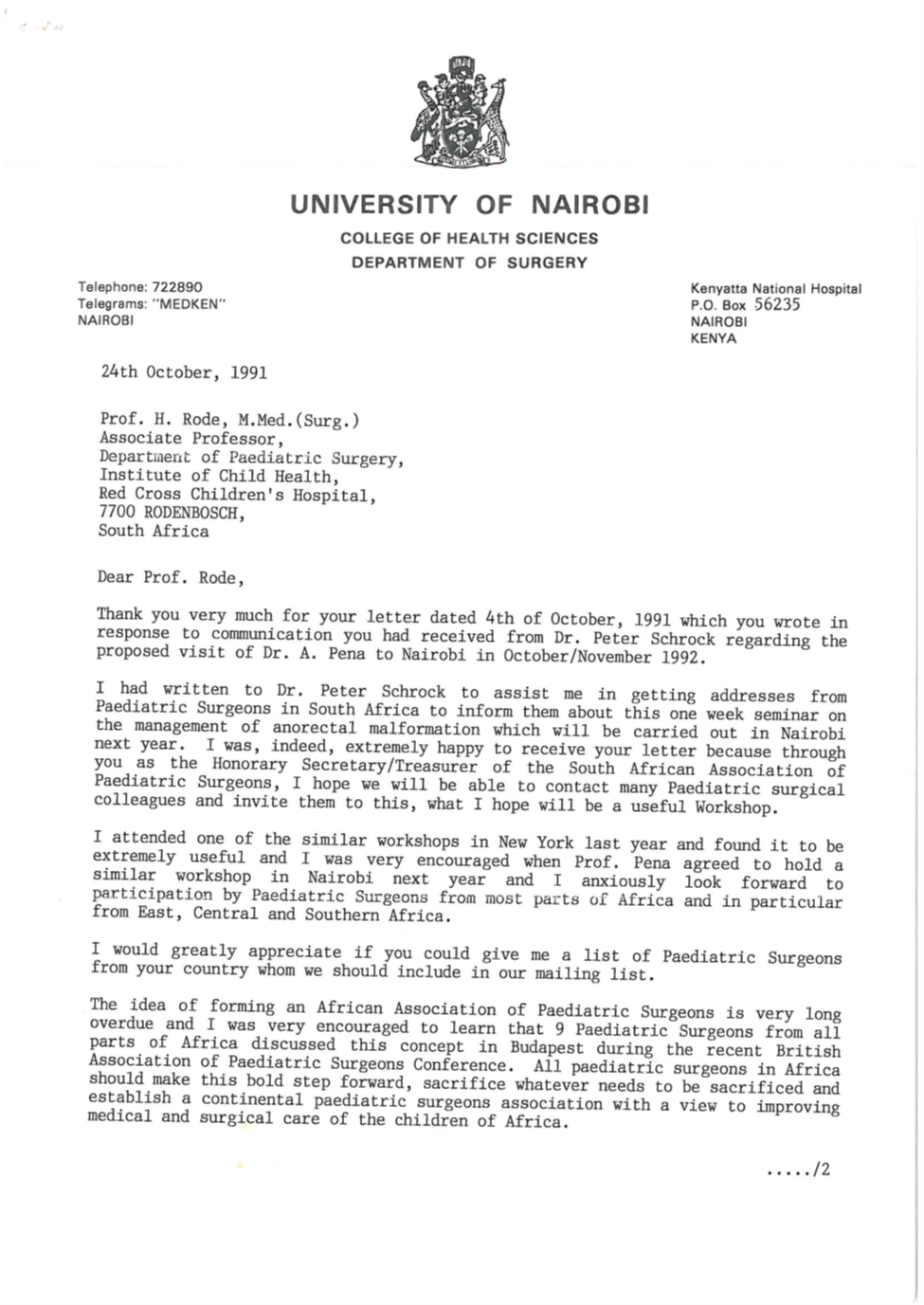


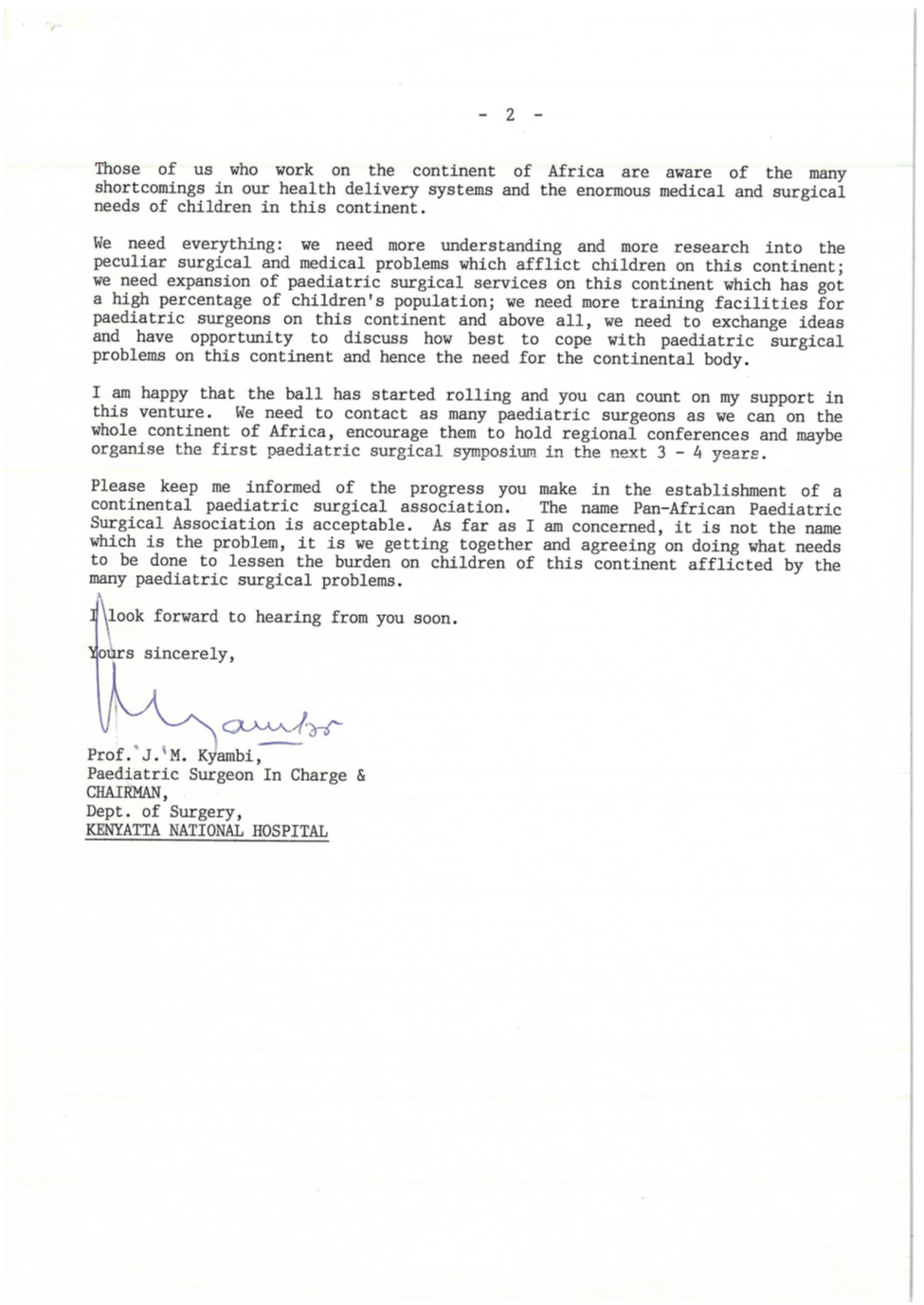

Supplement: Supplementary file 5 — Supplementary material 5 (DOCX 1419 KB) [file 383_2018_4248_MOESM5_ESM.docx]

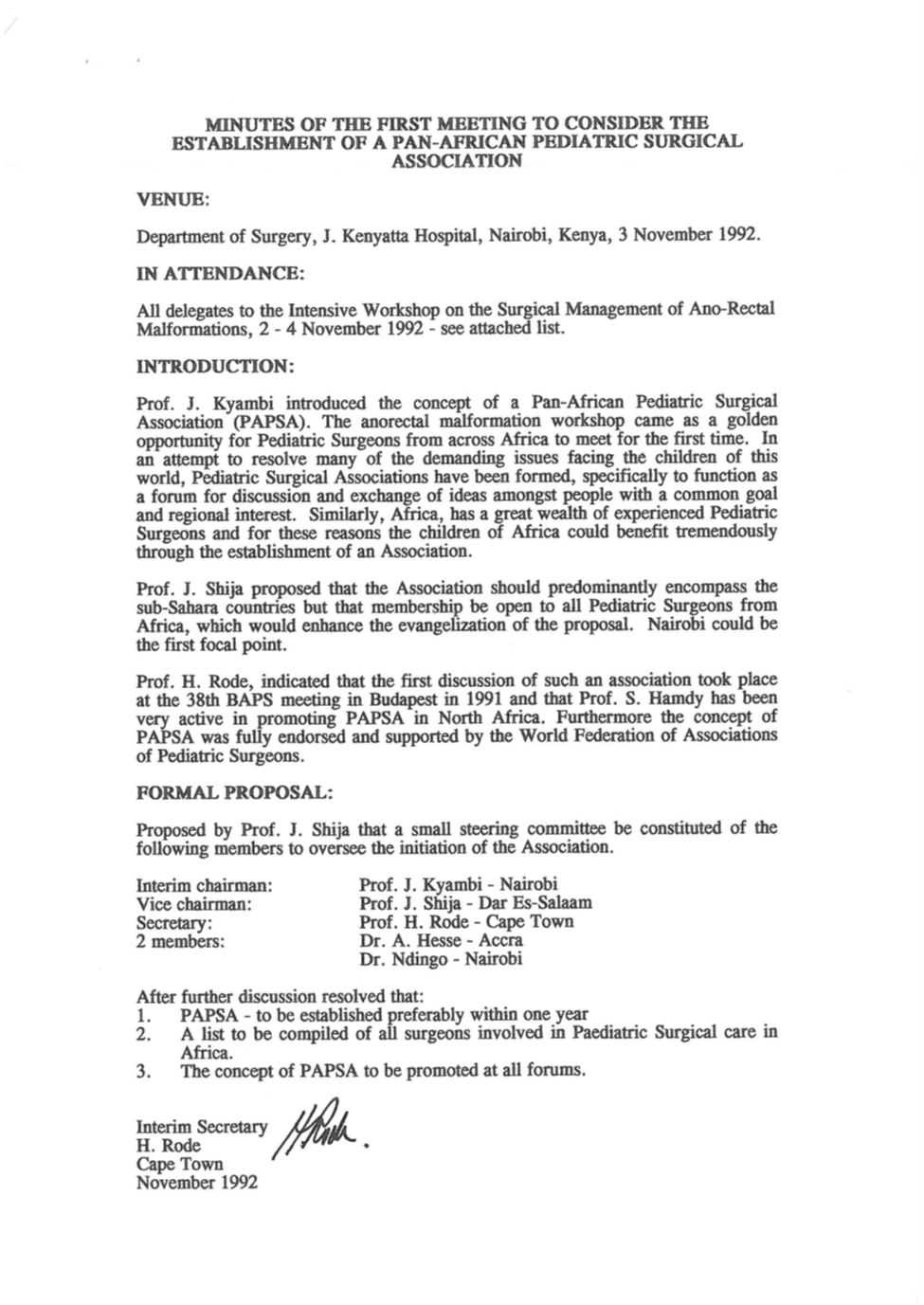

Supplement: Supplementary file 6 — Supplementary material 6 (DOCX 913 KB) [file 383_2018_4248_MOESM6_ESM.docx]

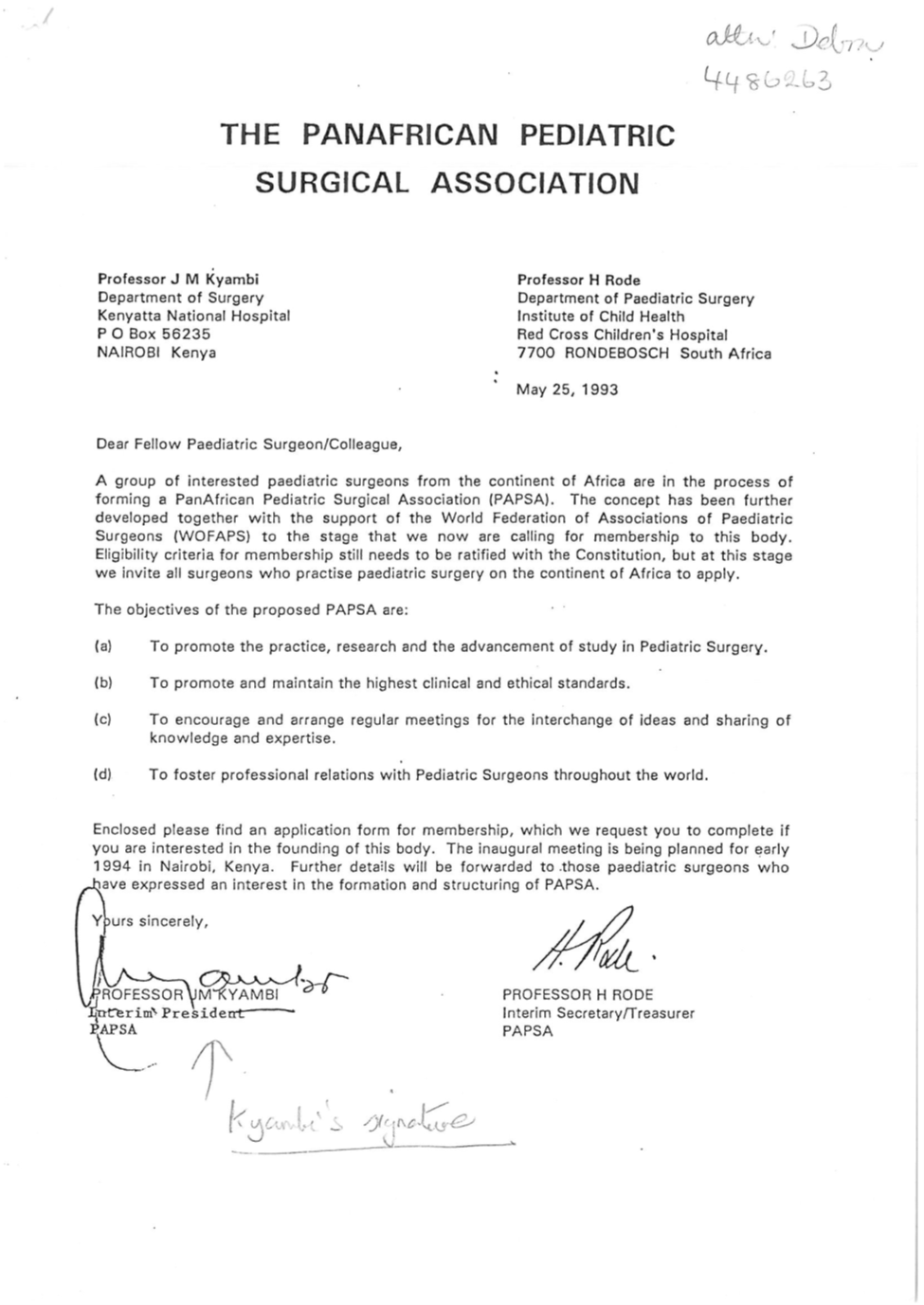

Supplement: Supplementary file 7 — Supplementary material 7 (DOCX 672 KB) [file 383_2018_4248_MOESM7_ESM.docx]

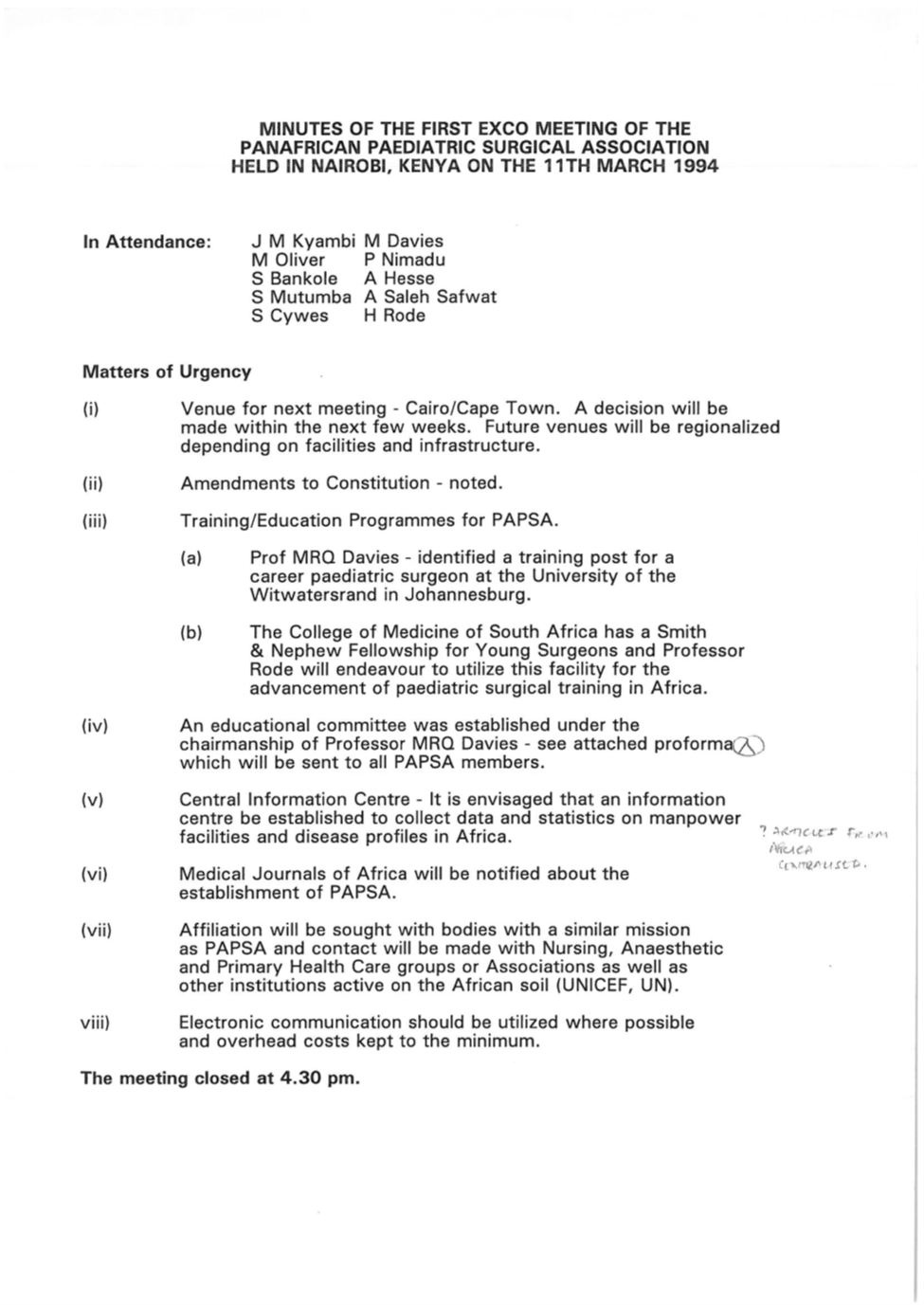

Supplement: Supplementary file 8 — Supplementary material 8 (DOCX 734 KB) [file 383_2018_4248_MOESM8_ESM.docx]

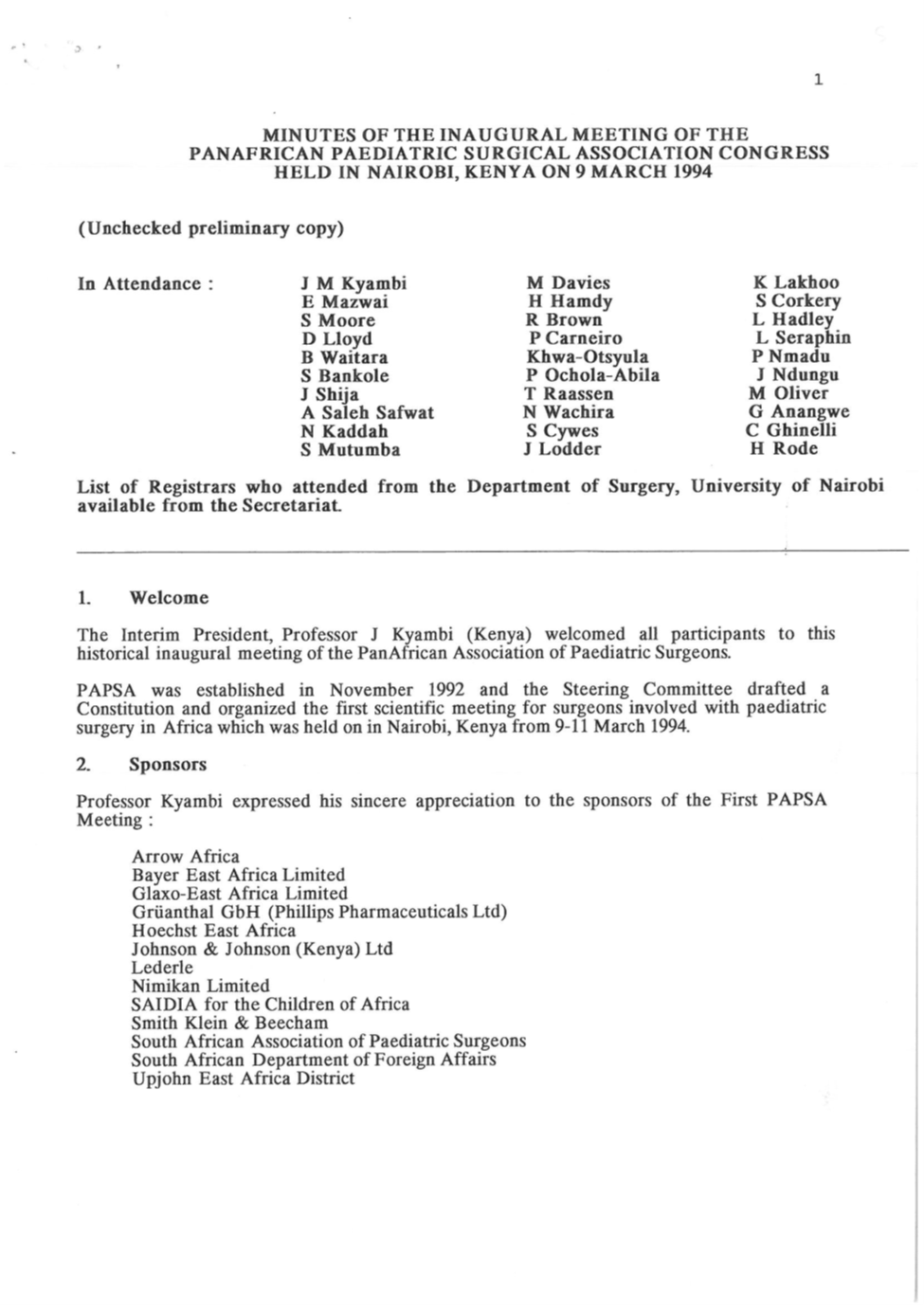


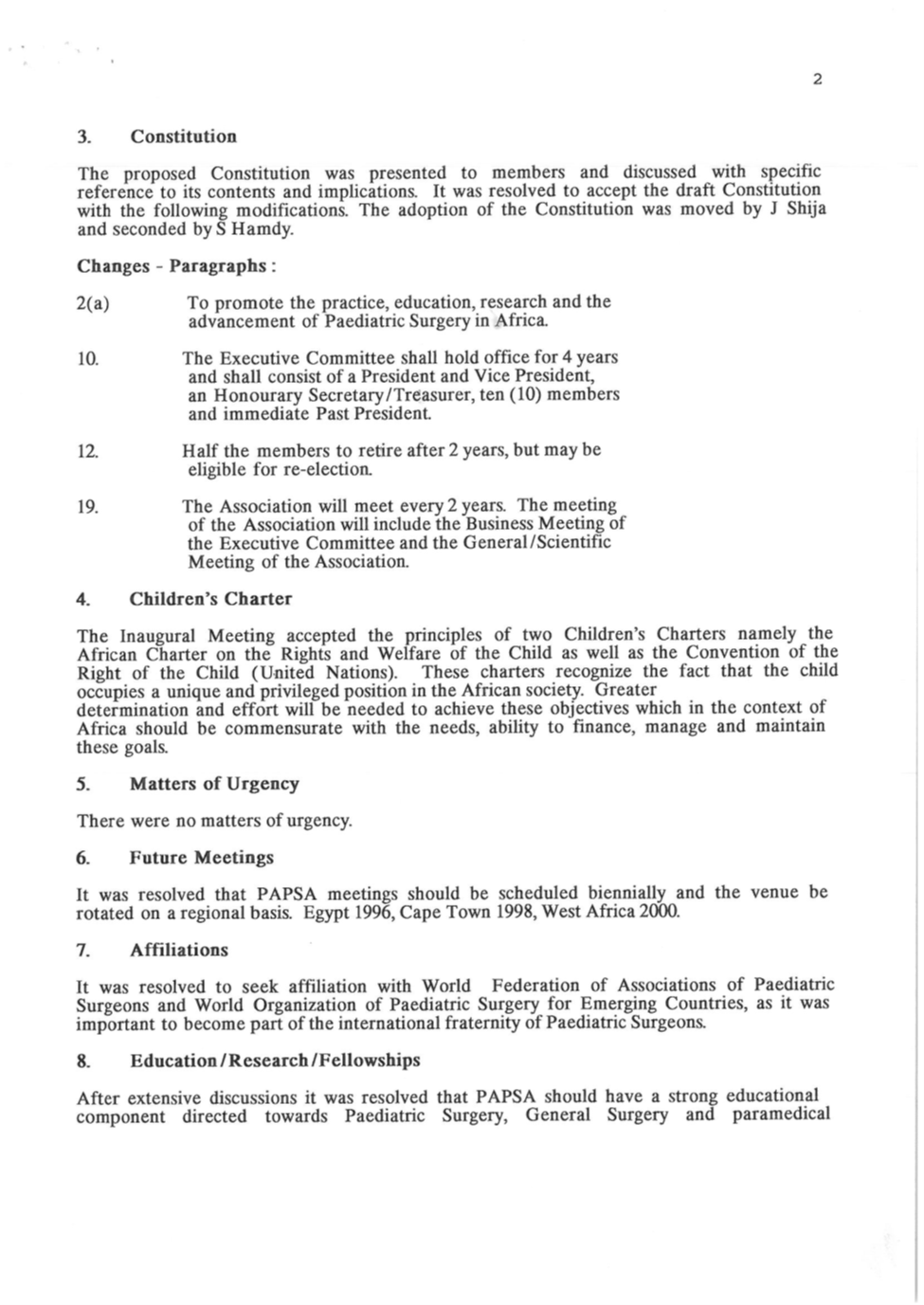


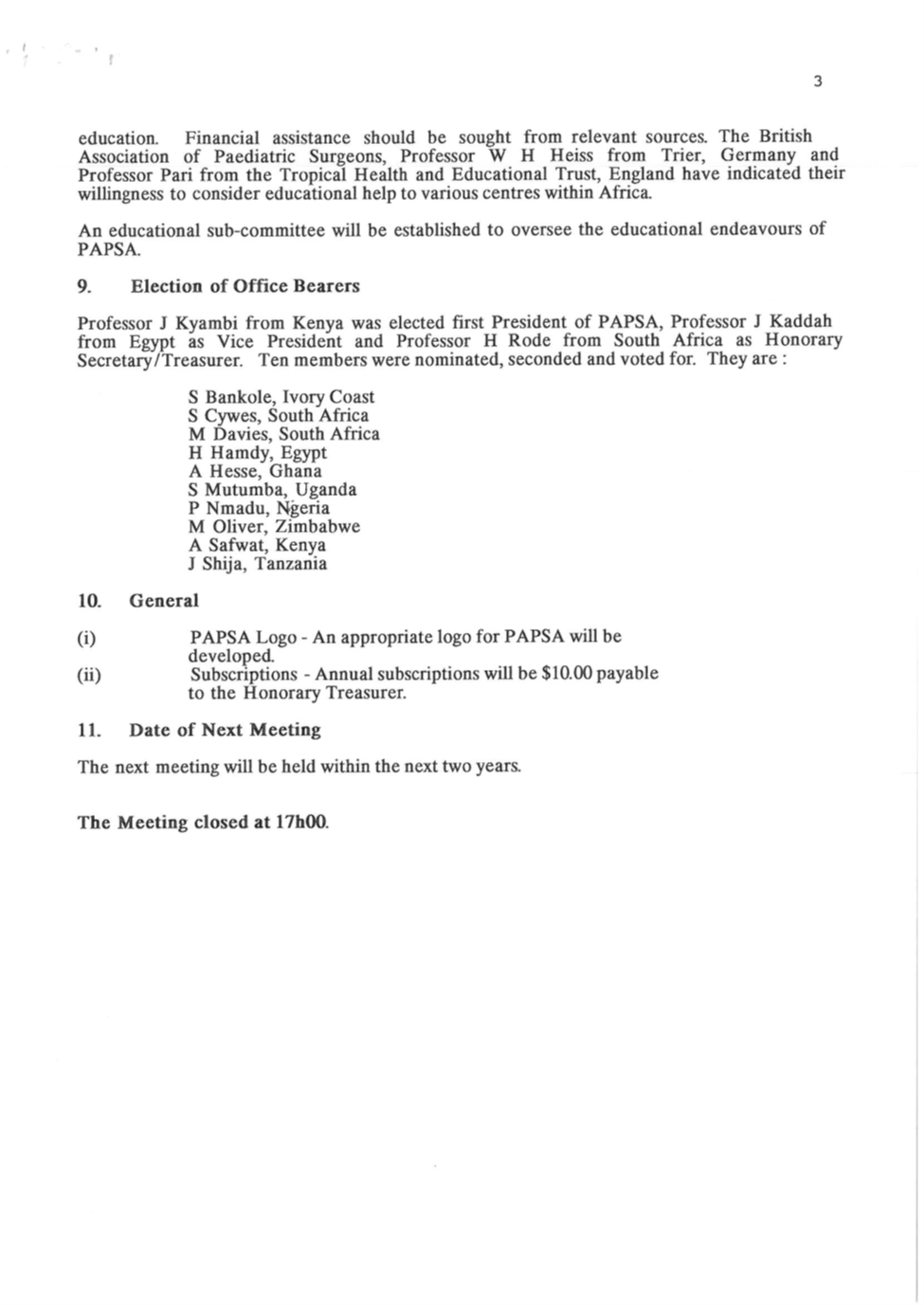

Supplement: Supplementary file 9 — Supplementary material 9 (DOCX 1122 KB) [file 383_2018_4248_MOESM9_ESM.docx]
